# Mental Health Professional Consultations and the Prevalence of Mood and Anxiety Disorders Among Immigrants: Multilevel Analysis of the Canadian Community Health Survey

**DOI:** 10.2196/19168

**Published:** 2020-09-16

**Authors:** Chinenye Nmanma Nwoke, Udoka Okpalauwaekwe, Hauwa Bwala

**Affiliations:** 1 Faculty of Health Sciences University of Lethbridge Lethbridge, AB Canada; 2 Department of Academic Family Medicine College of Medicine University of Saskatchewan Saskatoon, SK Canada; 3 Western College of Veterinary Medicine University of Saskatchewan Saskatoon, SK Canada

**Keywords:** immigrants, immigrant mental health, mental health consultations, mood disorders, anxiety disorders, mental health visits, Canadian Community Health Survey

## Abstract

**Background:**

There is a significant body of evidence on the link between migration and mental health stressors. However, there has been very little research on the use of mental health services by immigrants in Canada. The prevalence of mental health professional consultations among immigrants, as well as its correlations, are not well understood and remain largely unknown.

**Objective:**

This study aims to examine how specialist mental health visits (to a psychiatrist) differ from general mental health visits (to a family doctor or general practitioner) from immigrants, when compared to visits from those born in Canada, in a nationally representative sample of Canadian adults. This study also examines which group—immigrant or Canadian-born—suffers more from depression or anxiety, 2 of the more common mental health conditions.

**Methods:**

We used data from the Canadian Community Health Survey (CCHS) between the years 2015 and 2016. The outcome variables included consultation with any mental health professional, consultation with a specialist (psychiatrist), and the prevalence of mood and anxiety disorders. The independent variable was immigrant status. Other variables of interest were adjusted for in the analyses. Multilevel regression models were developed, and all analyses were performed with Stata IC statistical software (version 13.0, StataCorp).

**Results:**

The prevalence of mood and anxiety disorders was significantly lower among immigrants compared with individuals born in Canada; the prevalence of mood disorders was 5.24% (389,164/7,422,773) for immigrants vs. 9.15% (2,001,829/21,885,625) for individuals born in Canada, and the prevalence of anxiety disorders was 4.47% (330,937/7,410,437) for immigrants vs. 9.51% (2,083,155/21,898,839) for individuals born in Canada. It is expected that individuals with a lower prevalence of mood or anxiety disorders would use mental health services less frequently. However, results show that immigrants, while less likely to consult with any mental health professional (OR=0.80, 95% CI 0.72-0.88, *P*<.001), were more likely to consult with a psychiatrist (OR=1.24, 95% CI 1.04-1.48, *P*=.02) for their mental health visits when compared to individuals born in Canada.

**Conclusions:**

The results of this study reveal an unusual discord between the likelihood of mental health professional consultations with any mental health professional and mental health visits with psychiatrists among immigrants compared to nonimmigrants in Canada. Mental health initiatives need to be cognizant of the differences in the associated characteristics of consultations for immigrants to better tailor mental health services to be responsive to the unique needs of immigrant populations in Canada.

## Introduction

There is an extensive amount of published literature on mental health conditions in Canada. However, there is a paucity of research on mental health among immigrant populations [1]. A literature review on mental health concerns among Canadian immigrants showed varying trends and patterns. One study noted that immigrants have fewer episodes of mood disorders compared with their native-born counterparts [2]. Contrarily, 2 peer-reviewed articles reported an increased prevalence of mental health conditions such as emotional problems, stress, and increased psychotic disorders among Canadian immigrants [3,4]. Enabling factors identified to potentiate mental health problems among immigrants include racial discrimination and immediate difficulties with settling [4,5]. These situations are made worse by the barriers (ie, poverty, experiences of discrimination, stigma, language) faced by immigrants trying to access mental health services [2].

Access to mental health services significantly impacts immigrants, who have been currently identified as one of the most underserved in the Canadian healthcare system [4]. Studies have shown that only 50% of Canadians with mood disorders and anxiety disorders receive adequate care, in addition to long wait times for counseling and therapy services [6,7]. Specifically, immigrant communities in Canada underutilize mental health services in comparison to national and provincial averages [6,8]. For example, immigrant populations in Toronto utilized mental health services significantly less than Canadian-born participants (6% for recent immigrants, 7% for longer-term immigrants, and 10% for Canadian-born participants) [6]. With the increasing influx of immigrants to Canada, addressing gaps in the provision of and access to mental health services, including mental health professionals, among immigrants presents a rapidly emerging challenge.

An understanding of the relationship between mental health professional consultation and its association with immigrant status is largely unknown. The 2015 pan-Canadian survey on mental health services highlighted that 91% of survey respondents indicated that increasing access to mental health care professionals was a top priority for government action [9]. Examining this relationship is essential to identify potential gaps that need addressing within the mental health care system. Furthermore, understanding the relationship between mental health professional consultations and immigrants could give rise to strategies that would mitigate mental health–induced burdens associated with immigrant transition and assimilation into Canadian culture. More assuredly, a better understanding of immigrant mental health consultation patterns and mental health outcomes encourages patient-centered care in delivering mental health services to Canada’s booming immigrant population. The purpose of this study is two-fold and aims to answer the following research questions: (1) What is the variation of past-year mental health consultations (with general health practitioners and with psychiatrists) among immigrants compared to individuals born in Canada? (2) What is the prevalence of mood and anxiety disorders (2 of the most common mental health conditions) among immigrants compared to individuals born in Canada?

## Methods

### Data Source

We obtained data for the study from the 2015-2016 Canadian Community Health Survey (CCHS) [10]. The CCHS is an ongoing, national, cross-sectional survey that collects information on health status, health care utilization, and health determinants for the Canadian population. CCHS employs a cross-sectional study design to collect information related to health status and covers approximately 98% of the Canadian population aged 12 years and over. The CCHS data structure consists of individuals nested within health regions nested within provinces. CCHS data are collected using computer-assisted in-person and telephone interviewing. Further details on the methodology utilized by the CCHS and its measures can be found on the Statistics Canada webpage [10]. The CCHS uses a multistage stratified cluster design to sample household dwellings of the Canadian population aged 12 years and older living in private households. Excluded from the sampling frame were individuals living on First Nations reserves, institutions, remote regions, and full-time members of the Canadian Forces.

### Independent Variable

The immigrant sample, categorized as a binary variable (*yes* or *no*), is defined by Statistics Canada as “persons residing in Canada who were born outside Canada, excluding temporary foreign workers, Canadian citizens born outside Canada, and those with student or working visas” [11]. Participants in the CCHS were asked if they were born in Canada. Those that answered “no” to this question were considered immigrants. Nonimmigrants in this study refer to persons who were born in Canada.

### Outcome Variables

We assessed and analyzed 4 different outcomes related to self-reported responses to past-year mental health consultations. The questions included the following: (1) In the past 12 months, that is, from (date, one year ago) to yesterday, have you seen or talked to a health professional about your emotional or mental health? (*yes* or *no*) (2) Whom did you see or talk to? (Psychiatrist? *yes* or *no*) (3) Do you have a mood disorder such as depression, bipolar disorder, mania, or dysthymia? (*yes* or *no*) (4) Do you have an anxiety disorder such as a phobia, obsessive-compulsive disorder, or a panic disorder? (*ye*s or *no*). Respondents were to self-report conditions diagnosed by a health professional; however, they were not asked to elaborate on the type of mood disorder diagnosis or the time when the diagnosis occurred. All missing values for the outcome variables were dropped from the dataset during the statistical analyses.

### Other Covariates

Other covariates of interest adjusted for in the analysis include sex (male, female), age, marital status (married/common-law, not married/never married), sense of belonging (strong, weak), working status (not employed, employed), income adequacy (low income, income adequate), highest level of education (high school graduate or less, some post-secondary or more), owned or rented home, cultural or racial origin (Caucasian, visible minority), physical activity level (active, inactive), current smoking status (smoker, nonsmoker), and the number of chronic conditions present (zero, 1 or more).

### Statistical Analysis

Individuals were nested within health regions, which were further nested within provinces. Multistage analyses consisting of both descriptive statistics and multilevel logistic regression models were employed to identify relationships among variables measured at the individual level as well as the amount of variation of the effect at the group level (provinces and health regions). Tests of model fit using Akaike information criterion values were done to identify the level of multilevel logistic regression modeling that best fits the data. The estimated prevalence rates of the 2 mental health consultations associated outcomes were calculated for immigrant populations. Multilevel logistic regression modeling was carried out on each of the outcomes. The covariates outlined were assessed as potential characteristics of “any past-year mental health consultation,” “past-year psychiatrist consultation,” “self-reported diagnosis of mood disorder,” and “self-reported diagnosis of anxiety disorder.” The confounding effects of control variables on the study outcome variables were assessed at each stage of the model building process; any variable that caused a change of 20% or more on the regression coefficient of the primary covariates of interest was considered a confounder and was hence retained in the model. We calculated an intraclass correlation coefficient to measure the amount of variation shared by members of the same geographical area and to help us quantify the heterogeneity between the health regions and provinces.

We applied population sample weights to the effect size estimates to make inferences to the Canadian population. We used bootstrap weights provided by Statistics Canada in the CCHS data file to account for the complex sampling design used to calculate the 95% confidence intervals or the prevalence estimates. Bootstrapping is a technique used to estimate the variance of a statistic. The level of significance was set at 5%, with two-way analyses in reporting odds ratios and 95% confidence intervals as well. All analyses were performed using Stata IC (version 13.0, StataCorp) [12].

## Results

### Descriptive Statistics

In the CCHS 2015-2016, 25.33% (weighted percentage; 7,438,691/29,372,184) of the Canadian population surveyed (N=29,372,184) identified themselves as immigrants, with 25.30% (1,659,753/6,561,120) being recent immigrants and 74.70% (4,901,367/6,561,120) being nonrecent immigrants. The results of the descriptive analysis in the CCHS 2015-2016 are displayed in Table 1. The results show that 8.78% (624,617/7,110,919) of immigrants consulted any mental health professional in the past 12 months, 20.00% (124,559/622,661) had consulted with a psychiatrist, 5.24% (389,164/7,422,773) had mood disorders, and 4.47% (330,937/7,410,437) had anxiety disorders. The immigrant sample was 49.07% (3,649,845/7,438,691) male and 50.93% (3,788,846/7,438,691) female. The majority of the immigrants were 60+ years old (2,017,299/4,662,122, 43.27%), married (4,807,589/7,405,342, 64.92%), had a strong sense of belonging in the local community (4,860,802/6,988,638, 69.55%), were employed (4,451,118/6,718,851, 66.25%), earned an adequate income (5,571,604/7,434,459, 74.94%), had some post-secondary education or more (4,932,147/7,306,626, 67.50%), owned their home (4,991,492/7,395,539, 67.49%), were a visible minority (4,732,248/7,340,648, 64.47%), were physically active (5,345,682/7,016,883, 76.18%), were nonsmokers (6,565,496/7,430,428, 88.36%), and had no chronic conditions (5,283,483/7,387,161, 71.52%). Sensitivity analyses showed that there were no significant differences between outcome responses retained for analysis and those excluded by listwise deletion.

**Table 1:** Summary statistics for mental health professional consultation, mental health outcomes, sociodemographic, socioeconomic status, and behavioral variables by immigrant status (N=29,372,184).

Outcome variables, n (%)^a^		Canadian born, n=21,933,493 (74.67%)	All immigrants, n=7,438,691 (25.33%)	*P* values	
**Consulted any mental health professional in the past year?**					< .001
	Yes	3,434,221 (16.07)	624,617 (8.78)		
	No	17,936,177 (83.93)	6,486,302 (91.22)		
**Consulted a psychiatrist in the past year?**					.38
	Yes	573,903 (16.77)	124,559 (20.00)		
	No	2,847,760 (83.23)	498,102 (80.00)		
**Prevalence of self-reported mood disorders?**					< .001
	Yes	2,001,829 (9.15)	389,164 (5.24)		
	No	19,883,796 (90.85)	7,033,609 (94.76)		
**Prevalence of self-reported anxiety disorders?**					< .001
	Yes	2,083,155 (9.51)	330,937 (4.47)		
	No	19,815,684 (90.49)	7,079,500 (95.53)		
**Sex**					
	Male	10,849,193 (49.46)	3,649,845 (49.07)	0.73	
	Female	11,086,248 (50.54)	3,788,846 (50.93)		
**Age category in years**					< .001
	<20	2,609,211 (16.98)	422,982 (9.07)		
	20-39	3,407,653 (22.18)	1,007,521 (21.61)		
	40-59	3,802,799 (24.75)	1,214,320 (26.05)		
	60+	5,545,249 (36.09)	2,017,299 (43.27)		
**Marital status**					< .001
	Married/common-law	12,089,338 (55.22)	4,807,589 (64.92)		
	Not/never married	9,805,033 (44.78)	2,597,753 (35.08)		
**Sense of belonging in the local community**					.65
	Strong	14,398,157 (68.09)	4,860,802 (69.55)		
	Weak	6,748,070 (31.91)	2,127,836 (30.45)		
**Working status last week**					.67
	Employed	13,217,420 (67.66)	4,451,118 (66.25)		
	Not employed	6,318,019 (32.34)	2,267,733 (33.75)		
**Income adequacy**					< .001
	Low income household	4,454,184 (20.32)	1,862,855 (25.06)		
	Income adequate	17,465,194 (79.68)	5,571,604 (74.94)		
**Highest level of education**					< .001
	High school grad or less	9,285,274 (42.86)	2,374,479 (32.50)		
	Some post-secondary/more	12,376,542 (57.14)	4,932,147 (67.50)		
**Home rented or owned**					< .001
	Owner	16,559,806 (75.81)	4,991,492 (67.49)		
	Rented	5,283,586 (24.19)	2,404,047 (32.51)		
**Cultural or racial origin**					< .001
	Caucasian	19,164,163 (92.69)	2,608,400 (35.53)		
	Visible minority	1,510,714 (7.31)	4,732,248 (64.47)		
**Physical activity level**					< .001
	Active	16,216,008 (82.30)	5,345,682 (76.18)		
	Inactive	3,486,779 (17.70)	1,671,201 (23.82)		
**Current smoking status**					< .001
	Yes	4,207,071 (19.19)	864,932 (11.64)		
	No	17,717,523 (80.81)	6,565,496 (88.36)		
**Chronic conditions**					.004
	No conditions	15,363,198 (70.53)	5,283,483 (71.52)		
	1 or more conditions	6,418,787 (29.47)	2,103,678 (28.48)		

^a^Canadian Community Health Survey (CCHS) frequency weights have been applied to sample prevalence rate calculations.

From the stratified analysis shown in Table 2, the 2 immigrant groups (recent and nonrecent) were significantly different in the distribution of the outcome variables and all covariates (*P*<.001). Of note is the fact that nonrecent immigrants consulted mental health professionals more frequently (8.95% vs. 8.32%; *P*=.02), consulted a psychiatrist more frequently (21.77% vs. 16.64%; *P*=.51), and had a higher prevalence of mood (5.96% vs. 3.43%; *P*<.001) and anxiety disorders (4.92% vs. 3.18%; *P*<.001).

**Table 2:** Summary statistics for mental health professional consultation and mental health outcomes by recency of immigration.

Outcome variables, n (%)	All immigrants, N=7,438,691 (25.33%)	Recent immigrants (0-9 years), n=1,659,753 (25.30%)	Nonrecent immigrants (10+ years), n=4,901,367 (74.70%)	*P* values^a^
Consulted any mental health professional in the past year	624,617 (8.78)	135,096 (8.32)	416,307 (8.95)	.02
Consulted a psychiatrist in the past year	124,559 (20.00)	22,396 (16.64)	90,359 (21.77)	.51
Prevalence of self-reported mood disorders	389,164 (5.24)	56,854 (3.43)	291,501 (5.96)	< .001
Prevalence of self-reported anxiety disorders	330,937 (4.47)	52,702 (3.18)	239,839 (4.92)	< .001

^a^Chi-square test for differences between recent (0-9 years) and nonrecent (10+ years) immigrant groups.

### Multilevel Logistic Regression Analysis

Tables 3 and 4 show the multilevel logistic regression results with their respective odds ratios and 95% confidence intervals, adjusted for the primary predictor and other covariates.

**Table 3:** Final multivariate multilevel models A and B for immigrant status and mental health professional consultations and psychiatric consultations among respondents in the Canadian Community Health Survey (CCHS) 2015-2016.

Variable		Model A outcome: any mental health professional^a^			Model B outcome: specialized mental health professional (psychiatrist)^a^		
		OR	95% CI	*P* value	OR^b^	95% CI	*P* value
**Immigrant status**							
	No	Reference	Reference	—	Reference	Reference	—
	Yes	0.80	0.72-0.88	<.001	1.24	1.04-1.48	.02
**Sex**							
	Male	Reference	Reference	—	Reference	Reference	—
	Female	2.08	1.97-2.20	<.001	0.85	0.76-0.96	.006
**Age in years**				<.001			.001
	<20	Reference	Reference	—	Reference	Reference	—
	20-39	1.07	0.93-1.23	.36	1.03	0.85-1.27	.74
	40-59	0.96	0.83-1.11	.56	1.24	1.02-1.50	.03
	60+	0.45	0.39-0.52	<.001	0.94	0.77-1.15	.56
**Marital status**							
	Married/common-law	Reference	Reference	—	Reference	Reference	—
	Not/never married	1.43	1.35-1.52	<.001	1.40	1.22-1.59	<.001
**Sense of belonging to the local community**							
	Strong	Reference	Reference	—	Reference	Reference	—
	Weak	1.46	1.38-1.54	<.001	1.39	1.24-1.55	<.001
**Working status last week**							
	Employed	Reference	Reference	—	Reference	Reference	—
	Not employed	1.36	1.28-1.45	<.001	—	—	—
**Income adequacy**							
	Income adequate	Reference	Reference	—	Reference	Reference	—
	Low income household	1.19	1.12-1.27	<.001	0.79	0.70-0.91	.001
**Highest level of education**							
	High school grad or less	Reference	Reference	—	Reference	Reference	—
	Some post-secondary/more	1.23	1.17-1.31	<.001	—	—	—
**Home rented or owned**							
	Owned	Reference	Reference	—	Reference	Reference	—
	Rented	1.26	1.18-1.34	<.001	1.21	1.06-1.38	.004
**Cultural or racial origin**							
	Caucasian	Reference	Reference	—	Reference	Reference	—
	Visible minority	0.54	0.48-0.61	<.001	—	—	—
**Physical activity level**							
	Active	Reference	Reference	—	Reference	Reference	—
	Inactive	0.92	0.86-0.99	.03	—	—	—
**Current smoking status**							
	No	Reference	Reference	—	Reference	Reference	—
	Yes	1.26	1.18-1.35	<.001	1.25	1.10-1.42	.001
**Chronic conditions**							
	No conditions	Reference	Reference	—	Reference	Reference	—
	1 or more conditions	1.40	1.32-1.48	<.001	1.15	1.02-1.29	.03

^a^Intraclass correlation coefficient for Model A is 1.08% and Model B is 1.91%.

^b^Adjusted for employment status, highest level of education, physical activity, and chronic conditions.

**Table 4:** Final multivariate multilevel models C and D for immigrant status and the prevalence of mood disorders and anxiety disorders among respondents in the Canadian Community Health Survey (CCHS) 2015-2016.

Variable		Model C outcome: mood disorders^a^				Model D outcome: anxiety disorders^a^				
		OR^b^	95% CI		*P* value		OR^c^	95% CI		*P* value
**Immigrant status**										
	No	Reference	Reference		—		Reference	Reference		—
	Yes	0.79	0.71-0.89		<.001		0.73	0.65-0.83		<.001
**Sex**										
	Male	Reference	Reference		—		Reference	Reference		—
	Female	1.88	1.76-1.99		<.001		1.93	1.81-2.06		<.001
**Age in years**				<.001					<.001	
	<20	Reference	Reference		—		Reference	Reference		—
	20-39	1.21	1.05-1.39		.009		1.04	0.92-1.18		.48
	40-5	1.59	1.39-1.82		<.001		0.79	0.70-0.89		<.001
	60+	0.77	0.67-0.88		<.001		0.40	0.36-0.45		<.001
**Marital status**										
	Married/common-law	Reference	Reference		—		Reference	Reference		—
	Not/never married	1.39	1.29-1.49		<.001		1.19	1.11-1.28		<.001
**Sense of belonging to local community**										
	Strong	Reference	Reference		—		Reference	Reference		—
	Weak	1.95	1.83-2.08		<.001		1.66	1.56-1.77		<.001
**Working status last week**										
	Employed	Reference	Reference		—		Reference	Reference		—
	Not employed	1.61	1.50-1.72		<.001		1.60	1.49-1.72		<.001
**Income adequacy**										
	Income adequate	Reference	Reference		—		Reference	Reference		—
	Low income household	1.55	1.44-1.67		<.001		1.39	1.29-1.50		<.001
**Highest level of education**										
	High school grad or less	Reference	Reference		—		Reference	Reference		—
	Some post-secondary/more	1.08	1.01-1.15		.02		—	—		—
**Home rented or owned**										
	Owned	Reference	Reference		—		Reference	Reference		—
	Rented	1.44	1.33-1.54		<.001		1.39	1.29-1.50		<.001
**Cultural or racial origin**										
	Caucasian	Reference	Reference		—		Reference	Reference		—
	Visible minority	0.51	0.44-0.59		<.001		0.63	0.54-0.72		<.001
**Physical activity level**										
	Active	Reference	Reference		—		Reference	Reference		—
	Inactive	—	—		—		—	—		—
**Current smoking status**										
	No	Reference	Reference		—		Reference	Reference		—
	Yes	1.67	1.55-1.79		<.001		1.74	1.62-1.87		<.001
**Chronic conditions**										
	No conditions	Reference	Reference		—		Reference	Reference		—
	1 or more conditions	1.74	1.63-1.86		<.001		1.73	1.61-1.85		<.001

^a^Intraclass correlation coefficient for Model C is 2.31% and Model D is 1.34%.

^b^Adjusted for physical activity level.

^c^Adjusted for the highest level of education and physical activity level.

#### Consulted Any Mental Health Professional (Model A)

Immigrants had lesser odds of consulting any mental health professional than nonimmigrants (OR 0.8, 95% CI 0.7-0.9). Even when predisposing (age groups and sex) and enabling characteristics (owned home, employment status, etc) were taken into account, the association between immigrant status and any mental health professional consultation persisted (Table 3, Model A). The sociodemographic variables showed that the odds of consulting any mental health professional were almost twice as high for females when compared to males (OR 2.1, 95% CI 2.0-2.2). However, not married and never married respondents had significantly higher odds of consulting any mental health professional than married individuals (OR 1.4, 95% CI 1.4-1.5). Interestingly, individuals that felt a very weak sense of belonging to their local communities (OR 1.5, 95% CI 1.4-1.5), individuals between the ages of 20-44 years (OR 1.1, 95% CI 0.9-1.2), individuals from low-income households (OR 1.2, 95% CI 1.1-1.3), individuals that lived in rental accommodations (OR 1.3, 95% CI 1.2-1.3), smokers (OR 1.3, 95% CI 1.2-1.4), and individuals with 1 or more chronic conditions (OR 1.4, 95% CI 1.3-1.5) all had significantly higher odds of consulting any mental health professional in comparison with their respective reference groups.

#### Consulted a Psychiatrist (Model B)

Immigrants had higher odds of consulting with a psychiatrist than their nonimmigrant counterparts (OR 1.2, 95% CI 1.0-1.5). When predisposing (age groups and sex) and enabling characteristics (marital status, owned home, employment status, etc) were taken into account, the association between immigrant status and consultation of a psychiatrist persisted (Table 3, Model B). The sociodemographic variables showed that females had lesser odds of consulting a psychiatrist than males (OR 0.9, 95% CI 0.8-1.0). In addition, not married and never married individuals had significantly higher odds of consulting with a psychiatrist than their married and common-law counterparts (OR 1.4, 95% CI 1.2-1.6). Similarly, individuals that felt a very weak sense of belonging to their local communities (OR 1.4, 95% CI 1.2-1.6), those that lived in rental accommodations (OR 1.2, 95% CI 1.1-1.4), current smokers (OR 1.3, 95% CI 1.1-1.4), those with 1 or more chronic conditions (OR 1.2, 95% CI 1.0-1.3), and those aged 45-64 years (OR 1.2, 95% CI 1.0-1.5) all had significantly elevated odds of consulting with a psychiatrist compared to their respective reference groups. Individuals from low-income households had lower odds of consulting a psychiatrist (OR 0.8, 95% CI 0.7-0.9).

#### Presence of Mood or Anxiety Disorder (Model C and Model D)

In examining 2 of the most common psychiatric disorders, Table 4 shows that the immigrants had significantly lesser odds of having mood disorders (OR 0.8, 95% CI 0.7-0.9) and anxiety disorders (OR 0.7, 95% CI 0.7-0.8). Females, younger age groups, not married and never-married individuals, individuals with a weak sense of belonging to the local community, unemployed individuals, current smokers, and those with 1 or more chronic conditions also had increased odds of having mood or anxiety disorders. Individuals from visible minority groups had lesser odds of having both a mood disorder (OR 0.5, 95% CI 0.4-0.6) and an anxiety disorder (OR 0.6, 95% CI 0.5-0.7). These associations were both significant (*P*<.001).

Significant results between health region and provincial variation were identified for all outcome variables. After completing all 4 final models, it was found that the proportion of group-level variability explained by the health region level and provincial factors included in the models ranged from 1-2% for the odds of positively consulting any mental health professional, consulting a psychiatrist, and the presence of either a mood or anxiety disorder. We used 2-tailed Wald tests with statistical significance set at *P*=.05 to determine the statistical significance of the determinants in the final models, and only statistically significant variables were included in the final models shown in Tables 3 and 4. Model diagnostics were assessed using the Akaike information criterion at each stage of the model building process to ensure model fit and to check for outliers. All residuals were shown to be within acceptable levels.

## Discussion

### Principal Findings

The main objective of this study is to examine differences in any mental health professional consultation and psychiatrist consultations among immigrant and nonimmigrant groups in Canada. This study also examines the prevalence of mood and anxiety disorders among immigrants to Canada compared to those born in Canada.

The study results revealed that Canadian-born populations were more likely to suffer from self-reported diagnoses of mood and anxiety disorders compared with immigrant populations in Canada. This finding is consistent with other Canadian studies [13,14] and studies done in the United States [15-17]. Ideally, it would be expected that individuals with a more significant burden of mood or anxiety disorders would use mental health services more often. However, we found that immigrants, when compared to Canadian-born populations, were less likely to consult with any mental health professional (eg, psychologists, social workers, general physicians) and were more likely to consult with a psychiatrist for their mental health visits. This suggests an apparent primary care gap in the services received by immigrants and could be an indication of the reported severity of mental health issues immigrants commonly face [18,19]. The implication of this gap is a delay in the reception of care, leading to further deterioration of mental health with increasing cost implications to the individual and health care system.

It is common knowledge that psychiatrists typically tend to handle more severe cases of mental health issues than family physicians or other health care providers. Additionally, there is evidence in the literature showing how challenges with language, stigma, and other difficulties prevent immigrants from promptly accessing the mental health care they need [20-23]. These observations, taken into considerations with our findings of increased use of psychiatrists’ services, give further credibility to our position of a visible primary care gap, which could be mitigated by promoting support for the mental health of immigrants at an early stage of illness through primary health care services.

An alternative explanation for the frequent use of psychiatrists over other primary care providers could be that immigrants are more aware of the services that psychiatrists provide based on traditional knowledge from their home countries, in comparison to mental health services offered by psychologists, social workers, and family physicians. It is also possible that our findings might have been skewed to the individual preference for specialist care (ie, psychiatrists) [24]; however, a closer look at this study attributed its findings to the local presence of a psychiatric hospital and an extensive network of community-based organizations involved in mental health.

Interestingly, when respondents were stratified by self-reported diagnosis of mood and anxiety disorders, immigrants were more likely to consult a psychiatrist for their mental health than any other mental health professional as compared to Canadian-born populations. These findings—along with the identified effect of a sense of belonging, marital status, living arrangement, and being from a visible minority immigrant group—provide additional support for the need to explore mental health service utilization. The observed risk factors associated with mental health professional consultations in this study overlapped with many of the determinants found in other literature [25-27]. This study found that immigrants from a visible minority population (ie, racialized immigrants) were much less likely to report mental health consultations. The stressors of migration and resettlement have profound mental health implications, including mental health professional consultations. Long wait times, language barriers, lack of culturally safe care, the proximity of mental health services and difficulties with transportation, challenges in finding timely child care, and lack of suitable e-mental health technology implementation have been cited as primary reasons for unmet mental health care needs [7,21,28]. More importantly, a number of cultural factors (ie, differences in language and ethnicity of care providers) related to shame, stigma, and preference for help-seeking from family members have been cited as possible reasons for why Chinese populations in Canada persistently report the lowest levels of mental health service utilization [1]. These gaps warrant further research inquiry.

A few studies have reported gender differences in mental health-related consultations to be often nonexistent [29-31]. In our study, we found that female immigrants were more likely to consult with any mental health professional and with a psychiatrist as compared with male immigrants. However, men were more likely to have an inadequate number of mental health consultations compared with women, which could be due to gender differences in accessing health care (ie, women being more likely to have and keep to regular medical appointments with their family physician) [32-34]. This could imply that the mental health of immigrant men may be worse than that of immigrant women; nonetheless, further research is necessary to corroborate this claim further.

Our study provides additional insight into the type of mental health professional consultation an immigrant is more likely to seek. It is also insightful in offering education about alternative sources of mental health care to immigrant populations in Canada. Additionally, this could be useful for policymakers and service providers in targeting mental health funds and programs to vulnerable groups. Ultimately, addressing this issue could lead to increased health-related quality of life, improved productivity in the workforce, and decreased cost of medical resources targeted towards immigrant mental health care. More research is warranted to explore further and to understand better the discordance between mental health professional visits and psychiatric visits in general among immigrant and Canadian-born populations in Canada. Even so, a more comprehensive version of the CCHS is needed, especially one that takes into account immigrant-specific measures of variables such as language barriers in accessing mental health care, mental health stigma, level of mistrust of mental health services, the availability of culturally safe mental health care, and preferred immigrant help-seeking behaviors for mental health. These will help researchers and clinicians to better assess how mental health promotion programs can be tailored to address the specific mental health needs of immigrant populations in Canada.

### Study Limitations

Limitations of this study were, for the most part, related to the use of CCHS data. First, there were a limited number of mental health-specific variables in the dataset. This fact, in addition to the dichotomy of almost all variable responses, made it impossible to explore some other factors (for example, the sex of mental health professionals, and reasons for not consulting any mental health professional). Second, the CCHS data, although almost representative of the Canadian population, does not have data for on-reserve Aboriginal populations. Further research of this highly vulnerable group could have been explored and contrasted to findings among immigrant groups, as both groups face similar barriers in accessing culturally appropriate health and mental health care services in Canada. Third, as with all epidemiological studies employing cross-sectional data, we cannot make inferences about the temporality of events. Fourth, diagnostic tests for all models showed residuals within acceptable levels, hence implying the absence of outliers and increasing the reliability of the model. However, caution should be applied due to the limitations in the CCHS publicly available dataset. Lastly, the CCHS has the potential for recall bias as it requires participants to remember past events rather than corroborating with medical records. In addition, the outcome variables of mental health consultation (any and psychiatric) were based on a single survey item.

Despite these highlighted limitations, the use of the CCHS for this analysis provided comparative data from a large Canadian sample, which allowed for inferences to be made about the Canadian population as a whole. These findings could be further explored in future research with adjustments to the survey tools over time, especially as it relates to the inclusion of culturally specific variables.

### Conclusions

This study found that the majority of immigrants who seek mental health care consult the specialist services of a psychiatrist. As psychiatrists are known to deal with more complex cases of mental health, there is a crucial need for open conversations on mental health between family doctors and immigrants, as this is usually their first contact with the Canadian health care system. An expansion of the care role of family doctors may be a possible avenue to pursue to improve mental health service access for immigrant populations. A weak sense of belonging was strongly associated with past-year mental health consultation for immigrant populations. Mental health initiatives and policies need to be embedded in immigrant settlement initiatives to ensure that immigrants are better connected to their local communities. These could help immigrants get the right information about mental health in a timely manner and ultimately improve overall health and wellbeing. Cross-sectoral and holistic approaches are thus needed to move towards the comprehensive provision of timely and culturally safe mental health care delivery. This study examined conventional mental health professional consultation. Further research is required to understand how immigrant populations use other unconventional mental health supports as they relate to help-seeking behaviors for mental health; these can include social support networks, faith-based support, family or friend support, and spiritual or cultural services.

## Abbreviations

CCHS: Canadian Community Health Survey
